# FOXP3^+ ^T_regs _and B7-H1^+^/PD-1^+ ^T lymphocytes co-infiltrate the tumor tissues of high-risk breast cancer patients: Implication for immunotherapy

**DOI:** 10.1186/1471-2407-8-57

**Published:** 2008-02-23

**Authors:** Hazem Ghebeh, Eman Barhoush, Asma Tulbah, Naser Elkum, Taher Al-Tweigeri, Said Dermime

**Affiliations:** 1 Tumor Immunology Section, King Faisal Specialist Hospital and Research Center, P.O. Box 3354, Riyadh 11211, Saudi Arabia; 2 Department of Pathology, King Faisal Specialist Hospital and Research Center, P.O. Box 3354, Riyadh 11211, Saudi Arabia; 3 Department of Biostatistics, Epidemiology and Scientific Computing, King Faisal Specialist Hospital and Research Center, P.O. Box 3354, Riyadh 11211, Saudi Arabia; 4 King Faisal Cancer Center, King Faisal Specialist Hospital and Research Center, P.O. Box 3354, Riyadh 11211, Saudi Arabia

## Abstract

**Background:**

Recent studies have demonstrated a direct involvement of B7-H1, PD-1 and FOXP3 molecules in the immune escape of cancer. B7-H1 is an inhibitory molecule that binds to PD-1 on T lymphocytes, while FOXP3 is a marker for regulatory T cells (T_regs_). We have previously demonstrated the association of B7-H1-expressing T infiltrating lymphocytes (TIL) with high-risk breast cancer patients while other studies reported the involvement of FOXP3+ T_regs _as a bad prognostic factor in breast tumors. Although the co-existence between the two types of cells has been demonstrated *in vitro *and animal models, their relative infiltration and correlation with the clinicopathological parameters of cancer patients have not been well studied. Therefore, we investigated TIL-expressing the B7-H1, PD-1, and FOXP3 molecules, in the microenvironment of human breast tumors and their possible association with the progression of the disease.

**Methods:**

Using immunohistochemistry, tumor sections from 62 breast cancer patients were co-stained for B7-H1, PD-1 and FOXP3 molecules and their expression was statistically correlated with factors known to be involved in the progression of the disease.

**Results:**

A co-existence of B7-H1^+ ^T lymphocytes and FOXP3^+ ^T_regs _was evidenced by the highly significant correlation of these molecules (*P *< .0001) and their expression by different T lymphocyte subsets was clearly demonstrated. Interestingly, concomitant presence of FOXP3^+ ^T_regs_, B7-H1^+ ^and PD-1^+ ^TIL synergistically correlated with high histological grade (III) (*P *< .001), estrogen receptor negative status (*P *= .017), and the presence of severe lymphocytic infiltration (*P *= .022).

**Conclusion:**

Accumulation of TIL-expressing such inhibitory molecules may deteriorate the immunity of high-risk breast cancer patients and this should encourage vigorous combinatorial immunotherapeutic approaches targeting T_regs _and B7-H1/PD-1 molecules.

## Background

It is widely believed that dysfunction in the immune system of cancer patients allows the tumor cells to escape a process termed immunosurveillance [1-3]. A T lymphocytes inhibitory molecule named B7-H1 (also called PD-L1) expressed by antigen presenting cells has been shown to induce T lymphocyte anergy and/or apoptosis after ligation to its T lymphocytes receptor PD-1 [4-7]. B7-H1 has been shown to be directly involved in the protection of cancer cells from activated T lymphocytes [8]. Indeed, the expression of this molecule and its receptor has been described in several malignancies [9-17] where a strong link between its expression by the cancer cells and the patient clinicopathological status has been demonstrated in some of these malignancies [9,11,13,16]. We have shown previously that B7-H1 (PD-L1) is expressed in the tumor tissues of 50% of breast cancer patients and its expression was significantly associated with some important prognostic factors linked to high-risk patients [18].

Regulatory T cells (T_regs_) are a subset of T lymphocytes that regulate the immune response by suppressing the proliferation and cytokines production of effector T lymphocytes [19,20]. T_regs _are thus important for protecting our body by suppressing auto-reactive T lymphocytes. FOXP3, a forkhead/winged helix transcription factor was found to be essential for the development and control of T_regs _[21,22]. It has been used recently as a biomarker and a prognostic factor for malignant human tumor as reviewed in [23]. A direct link between the presence of T_regs _and progression of ovarian carcinoma has been demonstrated where human tumor FOXP3+ T_regs _were found to suppress tumor specific immunity and contribute to reduced survival of these patients [24]. In breast cancer, an increase in T_regs _population both in peripheral blood and tumor tissues was also reported [25] and a recent study demonstrated a significant intratumoral infiltration of FOXP3+ T_regs _in high-risk breast cancer patients and those at risk of late relapse [26].

Compelling evidences indicate a key role for the involvement of B7-H1 molecule in the development of T_regs_. Induction of adaptive CD4^+^CD25^+ ^T_regs _which develop in the periphery from CD4^+^CD25^- ^naïve T lymphocytes (in contrast to natural T_regs _formed in the thymus) could not be formed in mice which are B7 -/- [27]. In addition, mice vascular endothelial cells were able to induce CD4^+^CD25^+^FOXP3^+ ^T_regs _only in the presence of B7-H1 [28]. Another study has demonstrated the involvement of B7-H1 as an essential element for the induction of T_regs _after intra-tracheal delivery of an alloantigen [29]. Furthermore, B7-H1 was found to affect the function of T_regs _in which its blockade decreased the inhibitory effect of such cells [30] and blockade of B7-H1/PD-1 interactions abrogated T_regs_-mediated immunoregulation [31]. Although these studies have demonstrated the interaction between B7-H1 molecule and T_regs _in animal models their co-existence in cancer patients was not evaluated. This is the first study to investigate the correlation between FOXP3+ T_regs _and TIL-expressing B7-H1 and PD-1 in breast cancer patients. We have shown that their co-infiltration is strongly associated with high-risk prognostic factors and those T lymphocytes expressing FOXP3, B7-H1 and PD-1 molecules are of different T lymphocytes subsets.

## Methods

### Patients and samples collection

This study was conducted in accordance with Helsinki Declaration and all patients signed a consent form approved by the Research Ethics Committee of King Faisal Specialist Hospital and Research Center (KFSH&RC). The study was approved by the Research Advisory Council (RAC) of KFSH&RC (RAC# 2030 034).

Breast cancer specimens were collected from primary tumors of 68 patients including the 44 patients reported in our previous study [18] (median age 44 years) who were seeking treatments and had to undergo surgery (breast conservative surgery or total mastectomy) at KFSH&RC from 2003 to 2006. From the selected patients, 6 patients were removed from the study as they had no detectable TIL. Normal breast tissues were also obtained from 2 healthy women undergoing a plastic surgery and designated as BP. Upon excision of tissues by a surgeon, an anatomical pathologist obtained sample of the tumor tissue, denoted T, and an adjacent normal breast tissue from the same breast having the tumor, denoted N. Tissues from both T and N were processed as described before [18]. Briefly, they were fixed in formalin and embedded in paraffin for routine histopathological analysis while other piece was snap frozen in liquid nitrogen, preserved at -80°C and sectioned using a cryostat.

### Immunohistochemistry

Routine formalin-fixed, paraffin-embedded hospital tests were evaluated by immunohistochemistry for Her/2neu, Estrogen receptor, and progesterone receptor status as described before [18] while immunohistochemistry staining of frozen tissue sections were carried out as follows:

#### (a) Single staining

B7-H1 and PD-1 detection were carried out as described previously on fresh cryogenic sections with slight modifications [18,32] in which a very brief fixation in 2% paraformaldehyde (Fisher scientific) in PBS at RT (4 minutes for B7-H1 and 10 minutes for PD-1) was initially applied to improve the morphology of section. Briefly, sections were incubated for 15 minutes in 0.3% hydrogen peroxide solution and 0.1% sodium azide (Sigma, Saint Louis, MO, USA). Sections were blocked with 10% goat serum (DAKO, Denmark) for 30 minutes followed by B7-H1 antibody (MIH1 clone, ebioscience, San Diego, CA, USA) or PD-1 antibody (J116 clone, ebioscience,) diluted at 1:50 overnight. After washing, sections were stained for 30 minutes at RT, with Labeled Polymer (EnVision+) horseradish peroxidase (HRP) detection kit (DAKO). Colors were developed using DAB (Novocastra, Newcastle upon Tyne, UK) or AEC (Sigma) and sections were counterstained with instant hematoxylin (Shandon, Pittsburgh, PA, USA).

#### (b) Double staining

Sections were washed in PBS, incubated with paraformaldehyde for 4 minutes and blocked in H_2_O_2 _and goat serum as described above. The primary and secondary antibodies were added as follows: For CD3/FOXP3, CD8/FOXP3, CD8/PD-1 or CD8/B7-H1 double staining the 2 primary antibodies from two different species, mouse anti-FOXP3 (236A/E7 clone, ebioscience) diluted at 1:100, mouse anti-B7-H1 (clone MIH1, ebioscience) diluted at 1:25 or mouse anti-PD-1 antibody (J116 clone, ebioscience) diluted at 1:50 were mixed with rabbit anti-CD3 (Dako) at 1:50 dilution or rabbit anti-CD8 (Abcam, Cambridge, UK) at 1:75 dilution, together in 10% human AB serum (Cambrex) and incubated for 2 h at RT or overnight at 4°C. Sections were washed 3 times in PBS and incubated for 30 minutes at RT with a secondary antibody: swine anti-rabbit AP (Dako) diluted at 1:50 in a ready-to-use polymer (Envision+, Dako) linked to HRP. After washing 3 times with PBS, substrates were added starting with fuschin red (Dako) followed by DAB (Novocastra). Slides were counterstained with instant hematoxylin (Shandon). FOXP3 and B7-H1 double staining were carried out as described above except the primary antibodies used were rabbit anti-FOXP3 (polyclonal, Abcam) at 1:500 dilution mixed with mouse anti-B7-H1 (clone MIH1, ebioscience) at 1:25 dilution and overnight incubation at 4°C. FOXP3 and PD-1 double staining were started with a single staining of PD-1 as before, however after color development with DAB, sections were boiled for 3 minutes in an antigen retrieval citrate solution (pH = 6.0) to remove previously attached antibodies. FOXP3 antigen was then stained with mouse anti-FOXP3 antibody (236A/E7 clone, ebioscience) diluted at 1:100 for 2 hours at RT followed by goat anti-mouse IgG1 AP (Southern Biotech, Birmingham, Alabama, USA) for 30 minutes at RT. The color for FOXP3 staining was developed with Fuschin Red (Dako) and sections were counterstained with instant hematoxylin (Shandon).

### Interpretation of stained tumor sections

The B7-H1 and PD-1 staining were scored by an anatomical pathologist (AT) as described previously [18]. FOXP3^+ ^T lymphocytes were scored in 5–10% increments as a percentage of total CD3+ staining cells in several high magnification fields. Intratumoral FOXP3^+^/CD3^+ ^cells were only considered and counted while intrastromal FOXP3^+ ^cells were disregarded. FOXP3^+ ^cells with moderate or high intensity (++ or +++) were considered T_regs _while weak staining (+) were ignored. Due to the co localization of the CD3 and PD-1 molecules in the T lymphocyte membranes, double staining of PD-1 or B7-H1 and CD3 were assumed based on single-staining of sequential slides. The cut off point for positive and negative for FOXP3, B7-H1 and PD-1 was 5% (5% of total CD3+ T-lymphocytes).

### Statistical Analysis

Statistical analyses were used to determine the association between the B7-H1 expression in TIL or FOXP3^+ ^T_regs _infiltration and the patients' clinico-pathological parameters. Nominal parameters were analyzed using the fisher exact test. Comparisons of the independent variables with the dependent variable were performed using a simple logistic regression analysis. Significance was defined as the probability of a type one error of < 5%, and 95% confidence intervals were included. The software package SAS was used for these analyses.

## Results

### Frequencies of T_regs_, B7-H1+ and PD-1+ T lymphocytes in normal and breast cancer tissues

**Figure 1:**
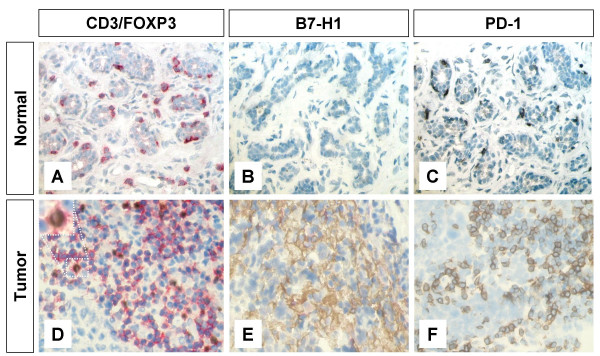
**Immunohistochemical staining of FOXP3+ T_regs _B7-H1 +and PD-1+ in T lymphocytes of breast tissues**. Representative micrographs at × 530 magnification of (A&D) CD3/FOXP3 double staining (red color, membranous for CD3 and brown nuclear color for FOXP3 expression). (B&E) B7-H1 single staining (brown color, membranous/cytoplasmic expression). (C&F) PD-1 single staining (brown color, membranous expression). Upper panel (A-C) is sections for normal breast duct and lower panel (D-F) is sections for infiltrating ductal carcinoma of the breast.

In order to determine the frequencies of T_regs_, B7-H1 and PD-1 expressing-T lymphocytes in normal breast tissues we stained sections obtained from breast patients undergoing plastic surgery (BP) as well as normal breast tissues adjacent to breast cancer tumors (N) for the expression of FOXP3, B7-H1 and PD-1 molecules. There were a few T lymphocytes in both N and BP in which FOXP3^+ ^T_regs _represented <5% of the total CD3^+ ^TIL (Figure 1A). Similarly, T lymphocytes in normal tissues were negative for B7-H1 (Figure 1B). However, PD-1+ T lymphocytes were abundant in normal tissues with up to 30% of CD3^+ ^cells co-expressing the PD-1 molecule (Figure 1C). In contrast to normal breast tissues, 56% of breast cancer patients had a higher T_regs _frequency in their tumor tissues in which 5–50% of CD3^+ ^TILs were T_regs _(Figure 1D). Similarly, B7-H1 was expressed in TILs of 54% of breast cancer patients' tumor tissues in which 5–80% of TILs express this molecule. B7-H1 expression was not restricted to T lymphocytes as 30% of breast cancer patients showed also B7-H1 expression in their tumor cells (Figure 1E). This result was also described in our previous study [18]. The PD-1 molecule was expressed in TILs in 60% of the patients in which 5–70% of TIL express this molecule (Figure 1F). The expression pattern of the PD-1 molecule was membranous and restricted to T lymphocytes only.

**Figure 2:**
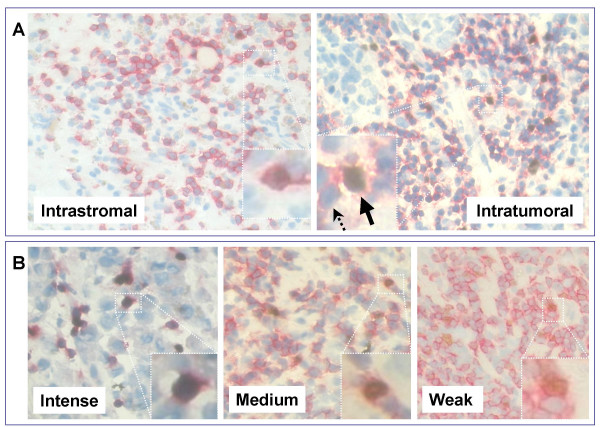
**Immunohistochemical staining showing intratumoral and intrastromal FOXP3+ T_regs _in breast cancer tissues**. Representative micrographs at × 530 magnification of CD3+ (red color membranous expression) and FOXP3+ (brown color nuclear expression) TIL of (A) intrastromal (left) and intratumoral (right) sections. Solid arrow indicates a CD3^+/^FOXP3^+ ^T_reg _cell and dashed arrow indicates a CD3^+^/FOXP3^- ^cell. (B) Intratumoral FOXP3^+ ^TIL with different staining intensity (Intense, Medium, and weak). Cells were counterstained with hematoxylin.

Both intratumoral and intrastromal FOXP3^+^CD3^+ ^cells were present in the patients tissues (Figure 2A) and the expression level of FOXP3 in the intratumoral T_regs _ranged from weak to intense (Figure 2B). Interestingly, most of FOXP3 intensely stained T_regs _were found in close proximity to tumor cells. PD-1^+ ^T lymphocytes were found both intrastromal and intratumoral, where they commonly seen in clumps, or as clumps/single cells respectively.

### Correlation of FOXP3, B7-H1 and PD-1 expression in TIL with clinicopathological parameters of patients

Breast cancer tissues infiltrated with intratumoral FOXP3+ T_regs _were found to be significantly associated with patients who had bad prognostic factors such as large tumor size (*P *= .029), histological grade III (*P *< .001), estrogen receptor negative status (*P *= .010) and severe lymphocytic infiltration (*P *= .051). Intratumoral FOXP3+ T_regs _were found also to be associated with progesterone receptor negative status and lymph node metastasis although they did not reach statistical significance (Table 1). The correlation of the expression B7-H1 in tumor cells and FOXP3+ T_regs _was also studied. There was a correlation between B7-H1 in tumor cells and FOXP3+ T_regs _(*P *< .019) (Table 1).

**Table 1:** Correlation of FOXP3^+ ^T_regs_, B7-H1^+ ^TIL and PD-1^+ ^TIL with the clinicopathological parameters of 62 breast cancer patients

	**FOXP3**			**B7-H1**			**◇PD-1**		
	**+**	**-**	*P	**+**	**-**	***P***	**+**	**-**	***P***
**Age**									
< 40 years	15 (68)^♣^	7 (32)	1.000	12 (55)	10 (45)	0.595	15 (68)	7 (32)	0.583
≥ 40 years	27 (68)	13 (33)		25 (63)	15 (34)		22 (58)	16 (42)	
									
**Tumor Size**									
<4 CM	18 (55)	15 (45)	**0.029**	16 (48)	17 (52)	0.072	17 (53)	15 (47)	0.187
≥ 4 CM	24 (83)	5 (17)		21 (72)	8 (28)		20 (71)	8 (29)	
									
**^◆^Lymph Node Metastasis**									
Negative	15 (75)	5 (25)	0.107	13 (65)	7 (35)	0.864	12 (63)	7 (37)	0.939
1 to 3	5 (42)	7 (58)		6 (50)	6 (50)		2 (29)	5 (71)	
4 to 9	12 (67)	6 (33)		11 (61)	7 (39)		12 (67)	6 (33)	
10 or more	9 (90)	1 (10)		6 (60)	4 (40)		5 (56)	4 (44)	
									
**Histological Grade (SBR)**									
II	14 (45)	17 (56)	**<0.001**	12 (39)	19 (61)	**0.002**	12 (40)	18 (60)	**0.001**
III	28 (90)	3 (10)		25 (81)	6 (19)		25 (83)	5 (17)	
									
**Lymphovascular Invasion**									
Positive	26 (70)	11 (30)	0.782	25 (68)	12 (32)	0.187	23 (64)	13 (36)	0.787
Negative	16 (64)	9 (36)		12 (48)	13 (52)		14 (58)	10 (42)	
									
Her2/neu status									
Positive	13 (62)	8 (38)	0.588	18 (62)	11 (38)	0.798	17 (61)	11 (39)	1.000
Negative	16 (64)	22 (58)		19 (58)	14 (42)		20 (63)	12 (38)	
									
ER status									
Positive	24 (57)	18 (43)	**0.010**	21 (50)	21 (50)	**0.029**	19 (48)	21 (53)	**0.001**
Negative	18 (90)	2 (10)		16 (80)	4 (20)		18 (90)	2 (10)	
									
**PR status**									
Positive	17 (57)	13 (43)	0.103	15 (50)	15 (50)	0.195	11 (38)	18 (62)	**<0.001**
Negative	25 (78)	7 (22)		22 (69)	10 (31)		26 (84)	5 (16)	
									
**Neo-adjuvant Chemotherapy**									
With	23 (64)	13 (36)	0.584	20 (56)	16 (44)	0.600	23 (66)	12 (34)	0.591
Without	19 (73)	7 (27)		17 (65)	9 (35)		14 (56)	11 (44)	
									
**Lymphocyte Infiltration**									
Focal	14 (52)	13 (48)	**0.051**	12 (44)	15 (56)	**0.017**	12 (46)	14 (54)	0.076
Moderate	14 (74)	5 (26)		11 (58)	8 (42)		13 (68)	6 (32)	
Severe	14 (88)	2 (13)		14 (88)	2 (13)		12 (80)	3 (20)	
									
**B7-H1 expression of Tumor cells**									
Positive	17 (89)	2 (11)	**0.019**	17 (89)	2 (11)	**0.002**	15 (83)	3 (17)	**0.041**
Negative	25 (58)	18 (42)		20 (47)	23 (53)		22 (52)	20 (48)	

**Abbreviations**: (+ and -) are number of positive and negative patients, ^♣^Numbers between brackets are the percentages of patients, ^◆^Two samples had unknown LN+ status, ^◇^Two samples had unknown PD-1 status *P values in bold represent a significant data.

We have reported previously on the expression of B7-H1 in 50% of 44 breast cancer patients; and found its expression in TIL to be significantly associated with large tumor size, high histological grade III, Her2/neu positive status and severe lymphocytic infiltration [18]. We further examined the B7-H1 expression in TIL of 62 breast cancer patients and correlated its expression with the same prognostic factors. Interestingly, very similar correlation was recorded for B7-H1+ TIL, and this was significantly associated with high histological grade III (*P *= .002), estrogen receptor negative status (*P *= .029), and sever lymphocytic infiltration (*P *= .017). There was also a correlation with large tumor size with a borderline significance (*P *= .072) (Table 1). There was also a correlation between B7-H1 in tumor cells and B7-H1+ TIL (*P *< .002) (Table 1). Although PD-1 molecule was also expressed by T lymphocytes infiltrating normal breast tissues lacking B7-H1, its presence in tumor tissues is considered essential for the inhibitory effect of B7-H1 molecule which is abundant in the tumor tissues. Therefore, we tested whether PD-1 is concomitantly expressed in the same patients along with B7-H1+ TIL and FOXP3+ T_regs_. We have found that PD-1+ TILs were abundant in patients with high histological grade III (*P *= .001), estrogen receptor negative status (*P *= .001) and progesterone receptor negative status (*P *< .001) (Table 1). There was also a correlation between B7-H1 in tumor cells and PD-1+ TIL (*P *< .002) (Table 1).

**Figure 3:**
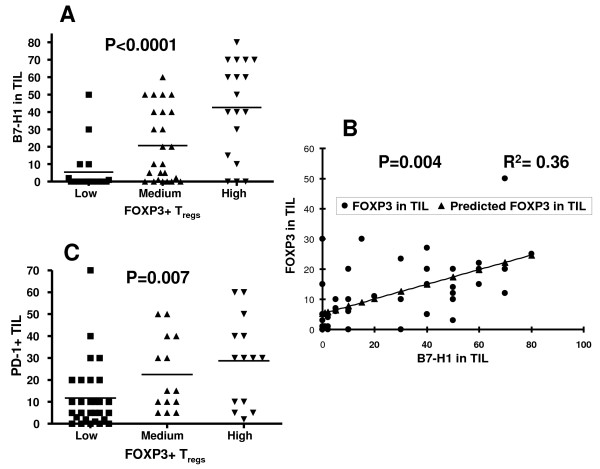
**Correlations between FOXP3+ T_regs_, B7-H1^+ ^TIL and PD-1^+ ^TIL**. (A) statistical analysis of FOXP3^+ ^T_regs _infiltration in tumor tissues with B7-H1^+ ^TIL, as analyzed with ANOVA of three groups of breast cancer patients with different FOXP3^+ ^T_regs _infiltration (Low = 0 to 4%, Medium = 5 to 14% and High 15% and above of total CD3^+ ^TIL), (B) their linear correlation analysis and (C) statistical analysis of FOXP3^+ ^T_regs _infiltration with PD-1^+ ^TIL in breast cancer as analyzed with ANOVA of three groups of breast cancer patients with different FOXP3^+ ^T_regs _infiltration (Low = 0 to 4%, Medium = 5 to 14% and High 15% and above of total CD3^+ ^TIL).

We have found that B7-H1+, PD-1+ TIL and FOXP3+ T_regs _were significantly associated with similar bad prognostic factors. Therefore, we further investigated the correlation between B7-H1+ TIL or PD-1+ TIL and FOXP3+ T_regs _infiltration in breast tumors. There was a highly significant correlation between the expression of B7-H1 in TIL and intratumoral FOXP3+ T_regs _(*P *< .0001) as shown by ANOVA test (Figure 3A) and by linear regression analysis (*P *< .004 and R^2 ^= 0.3) (Figure 3B). There was also a significant correlation between FOXP3+ T_regs _infiltration and PD-1 expression in TIL (*P *= .007) as shown by ANOVA (Figure 3C).

### Correlation of combined FOXP3, and B7-H1 expression in TIL with clinicopathological parameters of patients

We next tested whether a combination of FOXP3 and B7-H1 molecules will have a synergistic effect on the correlation with the patients' clinicopathological parameters. We used a combination of the two factors; FOXP3+ T_regs _and B7-H1 expression in TIL in the presence of PD-1 expression (only patients with B7-H1-expressing TIL were considered positive when PD-1+ TIL were present in the same tissue). The combined molecules maintained a significant correlation with the same prognostic factors (Table 2).

**Table 2:** Correlation between combined FOXP3+ T-reg and B7-H1 with the clinicopathological parameters of 62 breast cancer patients

	**FOXP3 and B7-H1 Expression**			*P
	**Both +**	**One +**	**Both -**	
**Age**				
< 40 years	21 (55)^♣^	7 (18)	10 (26)	0.496
≥ 40 years	10 (45)	7 (32)	5 (23)	
				
**Tumor Size**				
<4 cm	18 (64)	6 (21)	4 (14)	0.128
≥ 4 cm	13 (41)	8 (25)	11 (34)	
				
**^◆^Lymph Node Metastasis**				
Negative	12 (63)	3 (16)	4 (21)	0.605
1 to 3	4 (33)	3 (25)	5 (42)	
4 to 9	9 (50)	5 (28)	4 (22)	
10 or more	5 (56)	3 (33)	1 (11)	
				
**Histological Grade (SBR)**				
II	9 (30)	7 (23)	14 (47)	**<0.001**
III	22 (73)	7 (23)	1 (3)	
				
**Lymphovascular Invasion**				
Positive	20 (56)	8 (22)	8 (2)	0.745
Negative	11 (46)	6 (25)	7 (29)	
				
**Her2/neu Status**				
Positive	16 (57)	7 (25)	5 (18)	0.487
Negative	15 (47)	7 (22)	10 (31)	
				
**ER Status**				
Positive	16 (40)	10 (25)	14 (35)	**0.017**
Negative	15 (75)	4 (20)	1 (5)	
				
**PR Status**				
Positive	11 (38)	7 (24)	11 (38)	0.054
Negative	20 (65)	7 (23)	4 (13)	
				
**Neo-adjuvant Chemotherapy**				
With	17 (49)	9 (26)	9 (26)	0.828
Without	14 (56)	5 (20)	6 (24)	
				
**Lymphocyte Infiltration**				
Focal	10 (38)	5 (19)	11 (42)	**0.022**
Moderate	9 (47)	7 (37)	3 (16)	
Severe	12 (80)	2 (13)	1 (7)	

**Abbreviations**: (+ and -) are number of positive and negative patients, ^♣^Numbers between brackets are the percentages of patients, ^◆^Two sample had unknown LN+ status, *P values in bold represent a significant data.

### Expression of FOXP3, B7-H1 and PD-1 molecules by different subsets of TIL

**Figure 4:**
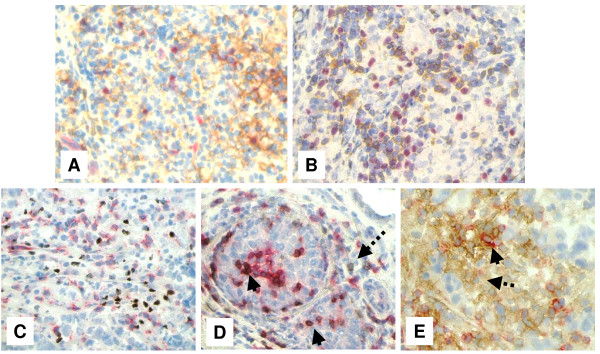
**Immunohistochemical staining showing the expression of FOXP3, B7-H1 and PD-1 molecules by different subsets of TIL**. Representative micrographs at × 530 magnification of (A) double staining of B7-H1 (brown color, membranous/cytoplasmic) and FOXP3 (red color, nuclear) in an area rich in TIL of tumor section. (B) Double staining of PD-1 (brown color, membranous) and FOXP3 (red color, nuclear) in sections from the same tumor as in A. (C) double staining of FOXP3 (brown color, nuclear) and CD8 (red color, membranous). (D) double staining of PD-1 (brown color, membranous) and CD8 (red color, membranous). Solid arrows indicate CD8^+^/PD-1^+ ^T lymphocytes and dashed arrow indicates a CD8^-^/PD-1^+ ^T lymphocyte. (E) Double staining of B7-H1 (brown color, membranous/cytoplasmic) and CD8 (red color, membranous). Solid arrow indicates a CD8^+^/B7-H1^+ ^T lymphocyte and dashed arrow indicates a CD8^-^/B7-H1^+ ^T lymphocyte.

We next asked whether FOXP3, PD-1 and B7-H1 molecules are expressed by different T lymphocyte subsets. Double-staining on 6 selected samples which had high FOXP3^+ ^T_regs_, high B7-H1^+ ^TIL and PD-1^+ ^TIL was performed. The selections were based on sections that had a good morphology to make the interpretation easier. Double-staining assays showed that B7-H1 and FOXP3 molecules were generally expressed by different T lymphocyte subsets (most B7-H1^+ ^TIL were FOXP3^- ^while only very few FOXP3^+ ^T_regs _co-express the B7-H1 molecule). In addition, the distribution pattern of B7-H1^+ ^TIL was different from that of FOXP3^+ ^T_regs_; T_regs _were single cells distributed over the section of the tumor tissues while B7-H1^+ ^TIL aggregated in clumps (Figure 4A). Similarly, PD-1 and FOXP3 molecules were expressed by different T lymphocyte subsets (Figure 4B). Furthermore, double staining assays demonstrated that FOXP3 and CD8 were mainly expressed by two different T lymphocyte subsets (Figure 4C) and only very few CD8+ TIL (< 5%) were FOXP3+. Therefore, it seems that most FOXP3+ T_regs _described here are of the CD4^+ ^subset since they were restricted to CD3^+ ^T lymphocytes. On the other hand PD-1 molecule was mainly expressed by CD8^+ ^T lymphocytes with only few CD8^- ^population were positive for PD-1 (Figure 4D). B7-H1 was mainly expressed by CD8^- ^T lymphocytes (Figure 4E) consistent with our previous observation of its expression by CD4+ T lymphocytes [18].

## Discussion

The immune system use intricate balance between positive and negative signals to protect the body from foreign agents while preventing autoimmunity [33]. This balance seems to be disturbed in various pathological conditions resulting in either inhibition of the immune system allowing invasion by tumor cells, or stimulation of the immune system to cause autoimmune diseases. For example, although interaction of B7-H1, a coinhibitory molecule, with its PD-1 ligand is important for prevention of autoimmunity [34], tumor cells use this interaction as a mechanism of immune evasion [35]. Furthermore, while the presence of T_regs _is important for suppression of host immune response to prevent autoimmune diseases [36], tumor cells can recruit T_regs _to inhibit anti-tumor immunity in cancer patients [37].

We have shown previously an abundant expression of B7-H1 molecule in high-risk breast cancer patients [18] who have highly proliferative tumor cells [32] and suggested the involvement of this molecule in the progression of the disease. In the present report we expanded our study to investigate the co-involvement of T_regs _with B7-H1 in the immune evasion of breast cancer. Although the interaction between the two types of cells has been demonstrated *in vitro *and animal models, their relative infiltration and correlation with the clinicopathological parameters of cancer patients have not been well studied. In this study, we investigated FOXP3+ T_regs _and TIL-expressing B7-H1 and its ligand PD-1 in breast cancer patients. We have used FOXP3 as a detection marker for T_regs _as it became recently a biomarker for studying T_regs _in malignant human cancers [23]. We have found that FOXP3^+ ^T_regs _are abundant in tumor tissues of 56% of breast cancer patients but absent in normal tissues adjacent to the tumors. FOXP3^+ ^T_regs _infiltrating tumors have been reported by other studies [25,26,38]. Abundant accumulation of T_regs _in tumor tissues might be due to the induction of CD4^+^CD25^+ ^T_regs _from peripheral CD4^+^CD25^- ^T lymphocytes [39,40] and/or migration of T_regs _from other parts of the body to the tumor area by chemotactic factors like CCL22 [24,41]. This hypothesis is supported by increase of T_regs _in peripheral blood of cancer patients [25,42].

Standard prognostic factors linked to high-risk breast cancer patients include: young age, large tumor size, high histological grade, positive lymph node metastasis and negative hormonal receptors status [43]. We have demonstrated that FOXP3^+ ^T_regs _infiltrating tumor tissues correlates significantly with important bad prognostic factors: large tumor size (*P *= .029), high histological grade (*P *< .001) and estrogen receptor negative status (*P *= .010). Similar findings have been recently reported by Bates *et al *showing an association between high T_regs _numbers, and high histological grade and estrogen receptor negative status [26]. Estrogen receptor negative breast tumors are generally associated with poor prognosis which is usually attributed to the association with high proliferative rate and lack of differentiation [44], that are features of progenitors of stem-like cells. Indeed it has been shown recently that stem cells obtained from both normal and cancer breast tissues lack the estrogen receptor [45]. Such stem cells were found to produce TGFβ1 [45] which is known to induce T_regs _[46]. The association of FOXP3^+ ^T_regs _with bad prognostic factors may suggest a contribution of T_regs _infiltrating breast cancer tissues to tumor escape from the immune system as their depletion lead to tumor rejection in animal models [47]. We have also confirmed the B7-H1 expression in TIL and its correlation with bad prognostic factors reported in our previous study [18]. The association of B7-H1 expression in TIL and FOXP3^+ ^T_regs _infiltration with the same bad prognostic factors suggests that both molecules correlate with each other. Indeed, there was a highly significant correlation between the expression of FOXP3^+ ^T_regs _and B7-H1 (*P *< .0001).

We have also found that PD-1 expression in TIL correlated with bad prognostic factors. PD-1 has been reported to be expressed in normal tissues to regulate self-reactive T cell responses [48]. In the present study PD-1 was expressed in up to 30% of T lymphocytes from normal breast tissues while up to 80% of TIL were PD-1^+^. The abundant expression of PD-1 in TIL together with B7-H1 (absent in normal tissues) seen in the present study may contribute to downregulation of the patients' immune response [4,6]. Furthermore, combination of FOXP3^+ ^T_regs_, B7-H1^+ ^TIL and PD-1^+ ^TIL were found to be highly associated with patients who had high histological grade III (*P *< .001) and estrogen receptor negative tumors (*P *< .007) suggestive of synergistic contribution to bad prognosis. It seems that FOXP3, B7-H1 and PD-1 molecules co-expressed in the TIL of the same tumor tissues may have synergistic effect in weakening the immune response of patients. IL-2 may play a fundamental role in regulating the interaction between FOXP3^+ ^T_regs_, B7-H1^+ ^TIL and PD-1^+ ^TIL. B7-H1/PD-1 interaction contributes to induction of T lymphocyte anergy [4] and this can be restored in the presence of IL-2 [49]. On the other hand, T_regs _are dependent on IL-2 for their expansion and function, and consumption of IL-2 present in the microenvironment may increase the inhibitory effect of B7-H1/PD-1 interaction [50].

We also investigated in the present study whether FOXP3, B7-H1 and PD-1 molecules are expressed by different T lymphocyte subsets. We have demonstrated that FOXP3+ T_regs _is a separate population from PD-1^+ ^and B7-H1^+ ^TIL. Similar results have been recently reported in non-Hodgkin's lymphoma by Yang *et al *[41] who showed that PD-1 and B7-H1 expression were mainly found in a subset of non-T_regs _T lymphocytes. The expression of FOXP3 was confirmed to be linked to CD4^+ ^T lymphocytes as it has been reported by Roncador *et al *[51]. We have also confirmed our previous findings showing that B7-H1 was expressed mainly in CD4^+ ^T lymphocytes [18]. On the other hand, we have shown that PD-1 molecules were expressed mainly by CD8+ T lymphocytes. It is important to mention that exhausted CD8^+ ^T lymphocytes have been reported to express the PD-1 molecule and its blockade restored the function of these T lymphocytes [52].

One of the most important findings of this study is the co-localization of immunosuppressive molecules expressed by different subsets of T lymphocytes in a group of high-risk breast cancer patients. Indeed, induction of immunosuppressive molecules such as B7-H1, PD-1 and T_regs _have been recently shown to counteract the anti-tumor effect of IL-12-based gene therapy in a transgenic mouse model of liver cancer [53]. Therefore, current interests in breast cancer immunotherapy should be focused on designing immunological tools to block B7-H1 and its PD-1 ligand and to deplete T_regs _in addition to cancer vaccination [54]. Blocking B7-H1 and PD-1 molecules with monoclonal antibodies or soluble ligands has been shown to enhance cancer immunity in animal models [55]. In addition, elimination of T_regs _by an anti-CD25 mAb enhanced anti-tumor immunity and induced tumor regression in animal models [47]. Furthermore, elimination of T_regs _by IL-2-conjugated to diphtheria-toxin (ONTAK) enhanced vaccine-mediated anti-tumor immunity in cancer patients [56]. Interestingly, a very recent study, in an animal model of renal cell carcinoma, has shown that only triple treatment consisting of tumor vaccine, B7-H1 blockade, and T_regs _depletion can result in a complete tumor regression and long lasting protective immunity [57] supporting the use of triple therapy treatment of cancer patients.

## Conclusion

In conclusion, we have shown a concurrent and abundant infiltration of different immune suppressive subsets of T lymphocytes in the microenvironment of high-risk breast cancer patients. This interesting observation suggests the development of a new therapeutic modalities aiming at targeting B7-H1/PD-1 and T_regs _in addition to still-developing immunotherapy.

## Competing interests

The author(s) declare that they have no competing interests.

## Authors' contributions

HG designed the study, carried out the immunohistochemistry for B7-H1, coordinated the work and wrote the manuscript. EB carried out immunohistochemistry of PD-1, FOXP3, and CD8 molecules. AT (anatomical pathologist) read and interpreted the sections. NE (statistician) carried out the statistical analysis. TA (medical oncologist) participated in conceiving the study and provided the clinical data. SD (principal investigator) wrote the proposal, conceived, supervised the study, and wrote the manuscript. All authors read and approved the final manuscript.

## Pre-publication history

The pre-publication history for this paper can be accessed here:


